# Equality in Educational Policy and the Heritability of Educational Attainment

**DOI:** 10.1371/journal.pone.0143796

**Published:** 2015-11-30

**Authors:** Lucía Colodro-Conde, Frühling Rijsdijk, María J. Tornero-Gómez, Juan F. Sánchez-Romera, Juan R. Ordoñana

**Affiliations:** 1 Murcia Twin Registry, Department of Human Anatomy and Psychobiology, University of Murcia, & IMIB-Arrixaca, Murcia, Spain; 2 Quantitative Genetics, QIMR Berghofer Medical Research Institute, Brisbane, Queensland, Australia; 3 MRC Social Genetic & Developmental Psychiatry Centre, Institute of Psychiatry, King’s College London, London, United Kingdom; 4 Department of Developmental and Educational Psychology, University of Murcia, Murcia, Spain; University of Reading, UNITED KINGDOM

## Abstract

Secular variation in the heritability of educational attainment are proposed to be due to the implementation of more egalitarian educational policies leading to increased equality in educational opportunities in the second part of the 20^th^ century. The action of effect is hypothesized to be a decrease of shared environmental (e.g., family socioeconomic status or parents’ education) influences on educational attainment, giving more room for genetic differences between individuals to impact on the variation of the trait. However, this hypothesis has not yet found consistent evidence. Support for this effect relies mainly on comparisons between countries adopting different educational systems or between different time periods within a country reflecting changes in general policy. Using a population-based sample of 1271 pairs of adult twins, we analyzed the effect of the introduction of a specific educational policy in Spain in 1970. The shared-environmental variance decreased, leading to an increase in heritability in the post-reform cohort (44 vs. 67%) for males. Unstandardized estimates of genetic variance were of a similar magnitude (.56 vs. .57) between cohorts, while shared environmental variance decreased from .56 to .04. Heritability remained in the same range for women (40 vs. 34%). Our results support the role of educational policy in affecting the relative weight of genetic and environmental factors on educational attainment, such that increasing equality in educational opportunities increases heritability estimates by reducing variation of non-genetic familial origin.

## Introduction

Changes in the heritability of educational attainment (EA) depending on situational conditions have long been a subject of interest to researchers from different disciplines. The analysis of such variations as related to educational policy may provide valuable information about the effects of the school system on individual differences in EA. It has been suggested that increasing equality in educational opportunities reduces variation of environmental origin and, consequently, leads to an increase in genetic variation (and thus the heritability) of this phenotype [1, 2]. That is, individual differences in the number of years of schooling may be attributed, broadly, to environmental factors, such as family socioeconomic status or availability of schooling facilities, or to individual traits with underlying genetic influences, such as intelligence, self-efficacy, personality or behavior problems [3]. As long as those environmental factors are similar for every individual (e.g., school facilities are available for everyone) they should not produce big differences in EA, and a higher proportion of individual differences in years of schooling should be attributed to genetic factors (heritability). In this sense, heritability could be viewed as an index of equity in educational opportunities [4]. This issue is not just related to an academic question. EA shows a direct relationship with socio-economic status, employability, occupation and income level [5, 6]. It is also a moderating variable for relevant aspects of human life, such as self-rated health, morbidity, disability and mortality; mental health and well-being; or quality of life [7–9].

The idea that more egalitarian educational policies increase heritability by means of a reduction in shared environmental variance has been pursued by different research groups. Heath et al. [10] found that in Norwegian males (but not females) born after 1940, the heritability increased (and shared-environmental effects decreased) as a possible effect of classical vs. more liberal social and education policies introduced after the Second World War. Tambs et al. [11] found a similar effect, also in Norwegian males born 1931–1935 and 1944–1960, interpreted as reflecting less social ascription affecting the second group. Still in Scandinavia (Sweden), Lichtenstein et al. [12] found a trend for shared environmental effects to be more important for EA in an older (above age 60) than in a younger cohort, while the genetic effects were more important in the younger cohort. However, these latter studies lacked sufficient power to draw firm conclusions.

Although family, twin and adoption studies, and even molecular genetics research, have provided consistent evidence for genetic variation in EA [12–18], the social change hypothesis has not found consistent support. Baker et al. [14] did not find differences in heritability between Australian birth cohorts born before and after 1950, nor between women and men, even when the sample was extended to much younger twins and different age thresholds were applied [19]. Other studies have tried to address this question using alternative approaches, like comparing the heritability of EA [20, 21] or analyzing the moderating effect of intelligence over genetic variance [22] between countries with, allegedly, differentially oriented educational policies.

**Fig 1:**
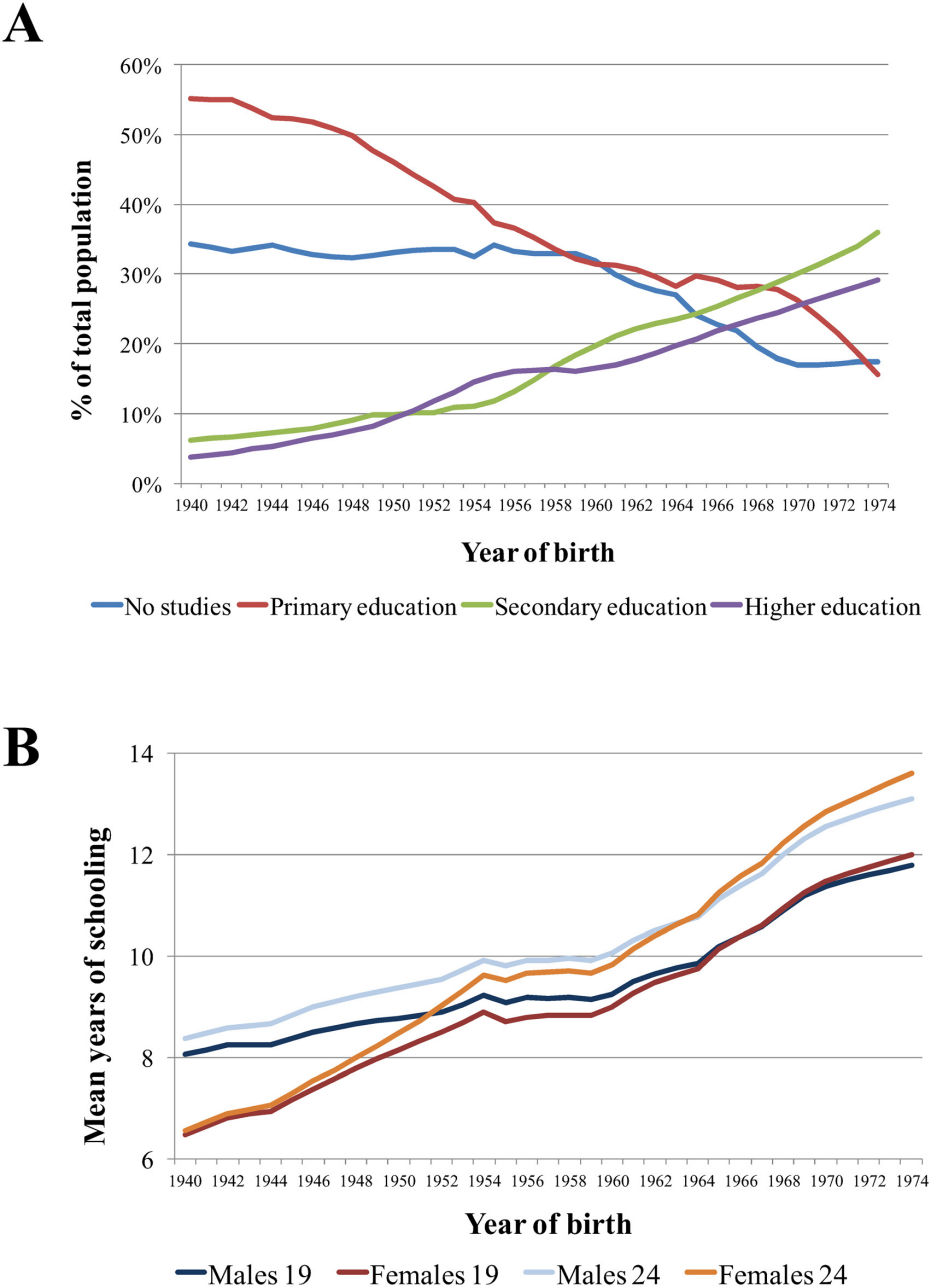
Evolution of the main indicators of Spain’s educational system from 1940 to 1974. (A) Educational attainment in Spain by year of birth. (B) Mean years of schooling by sex and year of birth for cohorts at 19 and 24 years old. Data source: *Historical Statistics of Spain*. *19* ^*th*^ *and 20* ^*th*^ *Centuries*. Fundación BBVA [23]

In spite of the interest of this question a definitive answer remains elusive and the role of educational policy on heritability estimations still generates controversy [2]. Part of this debate stems from the practical impossibility to isolate the specific effects of educational policy from the impact of its social milieu. The enquiry on this kind of topic is subject to restrictive assumption since no empirical analysis, in natural conditions, can separate the impact on EA of policy implementation and socioeconomic factors. Thus far, research on this topic has relied mainly on comparisons between countries with different educational systems or between time periods by using cut-points not directly related to changes in educational policies (e.g., pre- vs. post-war). Spain’s recent history offers an opportunity to incorporate new insights into this question, as the country experienced drastic changes in educational policy during the 20th century. One of the most important milestones during the second part of the century was the 1970 General Law on Education. This law marked an important change between the post-Spanish civil war education system and a more modern system which facilitated the adaptation of Spain to its geopolitical environment. Specifically, this law extended mandatory education up to 14 years old; a shift towards modern pedagogic approaches; the introduction of the same curricula for boys and girls; or the regulation of vocational training within secondary education. In general it was a landmark for the introduction of the principle of equality in educational opportunities within the Spanish educational system. Those changes, together with a global increase in the socioeconomic conditions in Spain, fostered the educational level of the country and increased EA for both genders [23]. Fig 1A and 1B shows some of the relevant changes that took place in EA from the early 1940’s to mid-1970’s in Spain. While the percentage of people with no studies remained stable since the end of the Spanish Civil War (1939), a decrease in this magnitude together with a rise in people accessing secondary or higher education starts for people born at the beginning of the 1960’s [23].

The first students under this reform were those born in 1961. This cut-point can be used to differentiate two cohorts drawn from the Murcia Twin Registry (MTR), which comprises twins born in the southeast of Spain between 1940 and 1966 [24]. Although, as mentioned before, reform effects cannot strictly be isolated from concomitant socioeconomic conditions, this circumstance provides an exceptional opportunity to study the effect of changes on educational policy on the heritability of EA. Our objective was to analyse two sub-samples of twins exposed to different educational systems in order to disentangle the genetic and environmental structure of EA variability under both periods. Regardless of the actual prevalence of EA before and after the reform, our hypothesis states that increasing equality in educational opportunities should increase the relative contribution of genetic effects (i.e. heritability) to the individual differences in EA, by means of a reduction of the influence of environmental factors on such differences.

## Materials and Methods

### Subjects and measures

A population-based sample of adult male, female and opposite-sex twins of the Murcia Twin Registry (MTR) [24] provided data for this study. The MTR comprises twin pairs born between 1940 and 1966 in the Region of Murcia, in the Southeast of Spain.

The sample selected for this study consisted of 2326 subjects (58% females). The mean age was 53.41 (SD = 7.1) for males and 54.25 (SD = 7.4) for females. The sample was distributed in 289 males from monozygotic twin pairs (MZM), 361 males from dizygotic same-sex pairs (DZM), 491 females from monozygotic pairs (MZF), 503 females from dizygotic same-sex pairs (DZF), as well as 327 males and 355 females from dizygotic opposite sex pairs (DOS). In total, the sample comprised 1271 pairs of twins (1055 complete and 216 incomplete). Zygosity was determined by questionnaire and DNA testing.

The data analyzed in this report were collected, by personal and telephone interview, in three different waves. Self-report data (years of education assessed by highest degree attained) was obtained. As participants were over 40 when they were interviewed, it was assumed that the reported EA should be definitive for nearly all cases; additionally, we presumed that no differences should be expected according to the moment of data collection.

EA ranged from Illiterate to University-High degree levels, following the guidelines of the Spanish National Statistics Institute. Achievement was categorized in: *low* (from Illiterate to completed Primary studies, being 45.6% of the sample), *medium* (completed General secondary education and Professional education basic degree, 32.5%) and *high* (from completed Superior secondary education to completed University-High degree, 21.9%).

Participants were divided into two groups: pre-reform cohort, consisting of those twins born before 1961 (65.4%) and the post-reform cohort, with those born in 1961 and after (34.6%).

### Ethics statement

All the participants in the MTR were previously contacted via an invitation letter containing the objectives of the registry and study information. Later on, they are contacted by telephone and oral informed consent is obtained prior to any data collection. When participants are involved in face-to-face interviews written informed consent is obtained. The MTR procedures, including informed consent, followed in this study have been approved by the Murcia University Ethical Committee and it follows national regulations regarding personal data protection. Applicable institutional and governmental regulations concerning the ethical use of human volunteers were followed during this research.

### Data analyses

Data preparation and descriptive and preliminary analyses were performed in SPSS v.19. Assumptions of the twin design (i.e., equal variances and means across birth-order and zygosity group) were checked by comparing a model with the applied constraints to a fully saturated model. Further details of the twin design, including assumptions, can be found elsewhere [25, 26].

**Fig 2:**
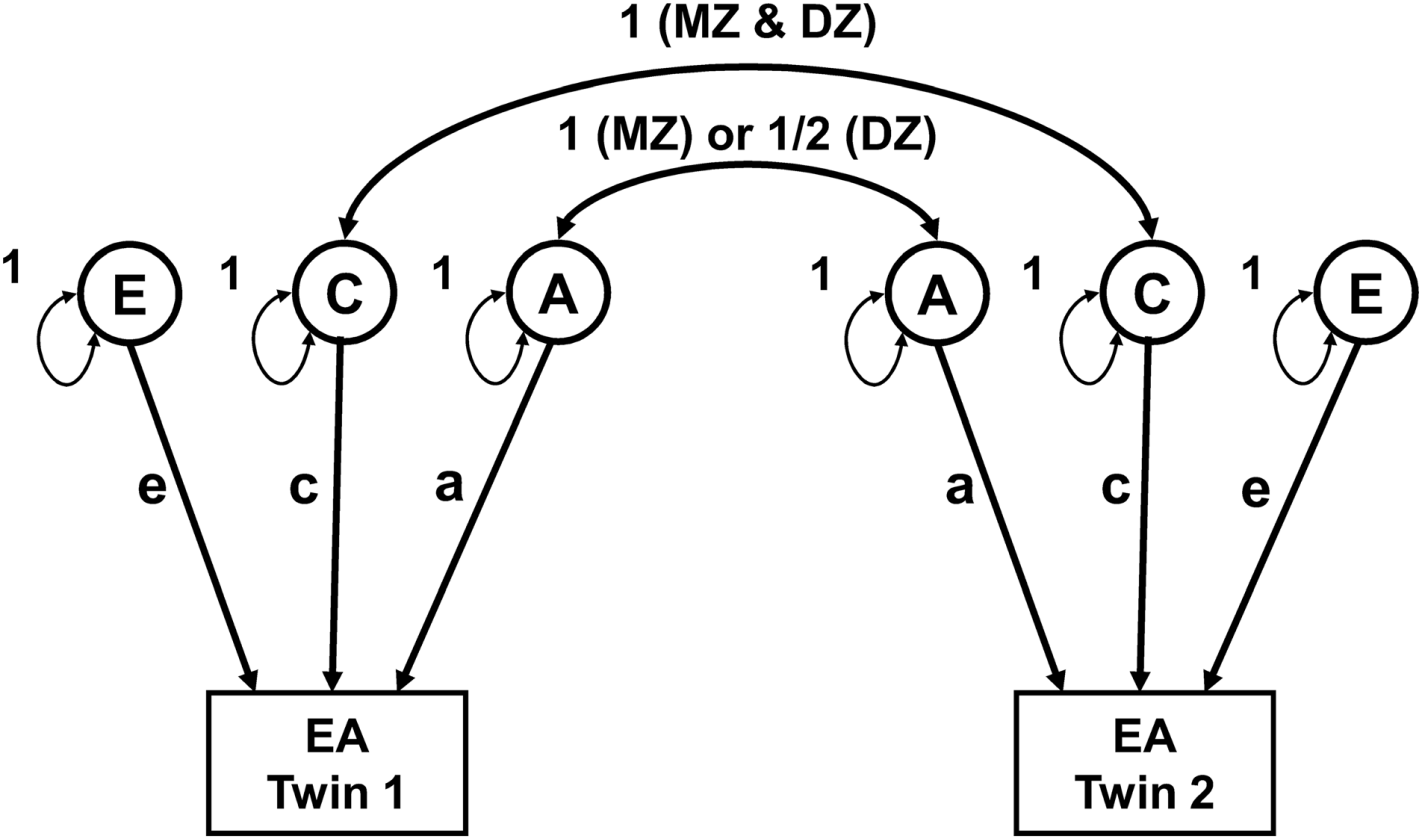
Path diagram for the basic univariate twin model used estimation of sources of variance in EA. The additive genetic factors (A) have a correlation of 1 between MZ twins and 0.5 between DZ twins, respectively. Shared family environment (C) is correlated 1 for both MZ and DZ twins. Unique environment (E) is the source of variance that will result in differences among members of one family and is, thus, uncorrelated between members of MZ and DZ pairs. The regression coefficients for A, C and E effects, are ‘a’, ‘c’ and ‘e’, respectively.

The data were analysed using Structural Equation Modelling (SEM), which allows one to test specific theoretical models in a multiple group approach. We applied the classical twin design [27, 28] to estimate the contribution of genetic and environmental factors to population variation in EA, using the statistical software package Mx [29]. Basically, the classical twin model compares intra-pair twin resemblance (twin correlations) between monozygotic (MZ) and dizygotic (DZ) twin pairs. The expectation of this difference forms the basis for estimating the contribution of latent variables defined as additive genetic factors (i.e. the sum of allelic effects across multiple genes) (A); common environment (i.e. environmental influences which make them similar, such as parental characteristics, intrauterine conditions, or family socioeconomic status) (C); and unique environmental factors (i.e. idiosyncratic experiences, as well as measurement error) (E). MZ twins share 100% of their segregating genes, while DZ share, on average, 50%. Hence the MZ correlation depends on their genetic makeup plus the effect of shared environmental factors (rMZ = A + C), while the DZ correlation is due to sharing half the genetic effects plus shared environmental factors (rDZ = ½ A + C). If MZ twins resemble each other significantly more than DZ twins, that is an indication of genetic effects on individual differences in a given trait. If DZ twins resemble each other more than half the resemblance of MZ twins, that is an indication of shared environmental effects. Non-shared environmental factors (E) are uncorrelated across individuals, and include measurement error (Fig 2).

Liability threshold models were fitted, assuming that the ordered EA categories reflect an imprecise measurement of an underlying normal distribution (with mean of 0 and variance of 1) and one or more estimated thresholds to discriminate between the categories [30]. This liability is further modelled to be influenced by genetic and environmental factors based on the MZ and DZ ratio in twin similarity which can be estimated by the correlations on the liability scale, called polychoric correlations. Following procedures described by Mehta et al. [31], when having more than 2 categories, threshold information can be used to estimate the mean and variance of the liability rather than fixing them, allowing homogeneity tests of variances across cohorts.

To be able to use all available data Full Information Maximum Likelihood estimation of raw data was used. In this method, twice the negative log-Likelihood (-2LL) of the data for each family is calculated, and parameters are estimated so that the likelihood of the data is maximized. Means, variances, and twin correlations were estimated in a saturated model. Equality in parameters across groups were assessed by likelihood ratio tests (LRT), obtained by subtracting -2LL of a restricted model from that of a full model. The resulting test statistic has a χ^2^ -distribution with degrees of freedom (*df*) equal to the difference in *df* between the two models. The best-fitting model was chosen in each case by deducting the residual deviance of the compared models and by comparing Akaike’s Information Criterion (AIC).

## Results

The distribution of our categorized EA variable according to sex and cohort is presented in Table 1. Two relevant facts are notable. First, there are clear cohort differences, so that individuals born after 1960 are more likely to have completed both medium and high education levels. Secondly, sex differences in these proportions show females to be less likely to achieve a high educational level regardless of cohort.

**Table 1:** Distribution of completed educational levels by sex and cohort.

	Pre-reform cohort (1940–1960)				Post-reform cohort (1961–1966)			
	*n*	Low (%)	Medium (%)	High (%)	*n*	Low (%)	Medium (%)	High (%)
Males	624	53.0	24.7	22.2	353	17.4	46.8	35.8
Females	897	62.2	25.0	12.8	452	25.7	48.1	26.2
Total	1521	58.5	24.9	16.6	805	22.2	47.5	30.3

*n*: number of observations.

Preliminary analyses comparing different models confirmed that means and variances were significantly different between cohorts for males and females separately, since equating means or variances between cohorts produced a significant loss of goodness-of-fit (p < .05) in all cases. Equating male and female means within cohorts also produced a similar worsening of fit (p < .05). However, variances for males and females within cohorts could be equated without significant loss of fit (Pre-reform: χ^2^ = 3.18; df = 1; p > .05; Post-reform: χ^2^ = 0; df = 1; p > .05).

Table 2 shows the polychoric correlations between cotwins, by zygosity group and birth cohort. A general trend for lower correlations in the post-reform cohort is observed, which suggests a more relevant role of unique environmental factors. Table 2 further shows that there are no sex differences in the genetic architecture of EA in cohort 1, since the MZ:DZ correlation ratios are very similar for males and females. However, these ratios change for males in cohort 2, but stay the same for females. This pattern suggests both appreciable cohort and sex differences in the relative effects of genetic and environmental factors on EA, pointing to a higher relative influence of genetic factors, at least for males born from 1961 onwards. Additionally, the DZO correlation is between that of DZM and DZF in both cohorts, suggesting there are not qualitative sex differences in genetic and environmental effects.

**Table 2:** Polychoric twin correlations with 95% Confidence Interval (CI) by zygosity, sex, and cohort.

	Pre-reform cohort (1940–1960)		Post-reform cohort (1961–1966)	
	*n* (pairs)	*r (95% CI)*	*n* (pairs)	*r (95% CI)*
MZM	91	.88 (.75,.94)	67	.75 (.48,.87)
DZM	118	.65 (.43,.79)	81	.33 (.01,.56)
MZF	149	.88 (.78,.93)	103	.80 (.64,.87)
DZF	179	.67 (.50,.79)	88	.62 (.39,.77)
DZO	293	.67 (.54,.76)	102	.42 (.09,.64)

*n*: number of observations;

MZM: monozygotic male pairs;

DZM: dizygotic male pairs;

MZF: monozygotic female pairs;

DZF: dizygotic female pairs;

DZO: dizygotic opposite sex pairs.

**Fig 3:**
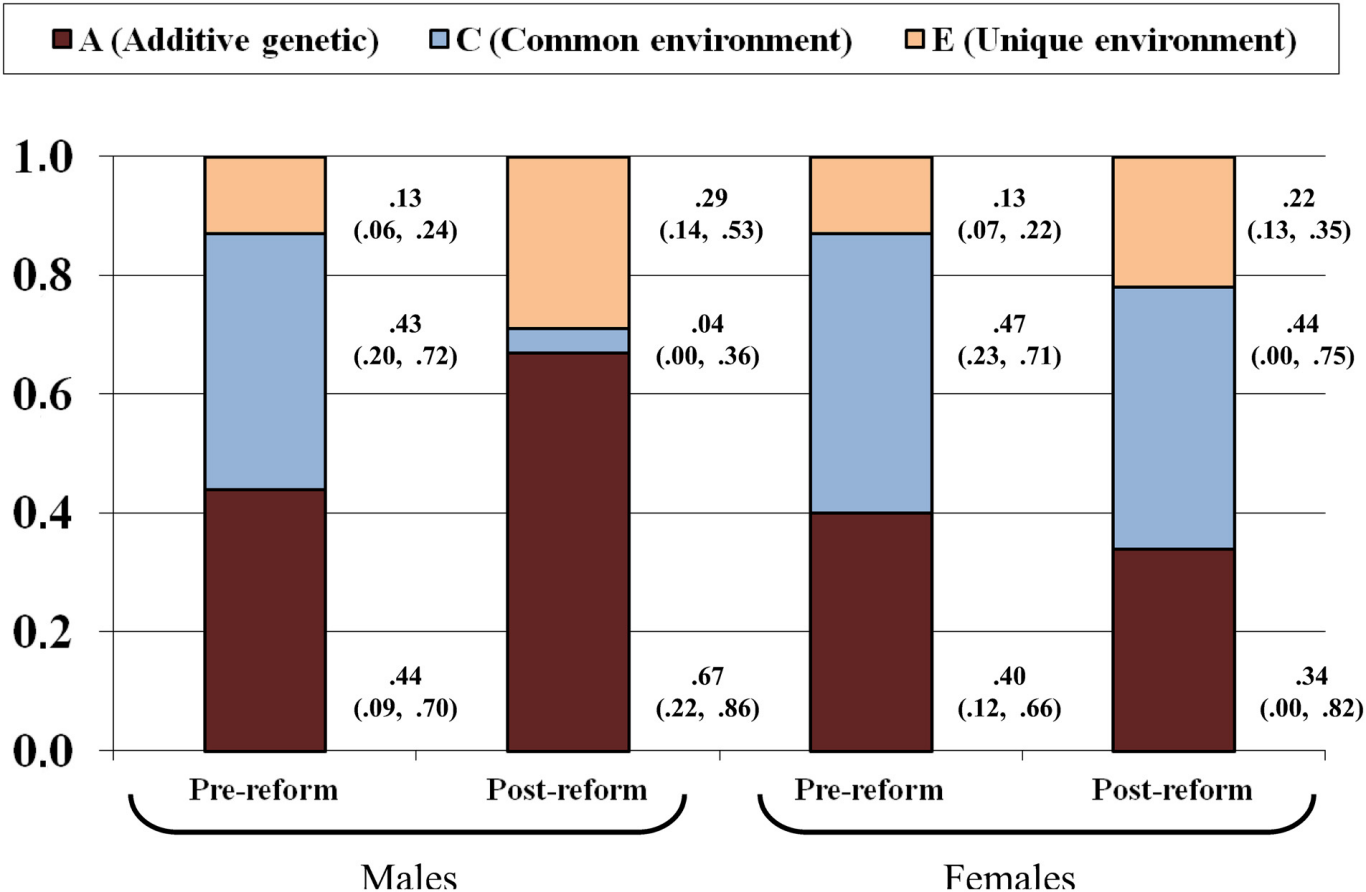
Proportions of variance explained (95% CI), by sex and cohort.

These observations were confirmed by model-fitting analyses. The proportions of variance explained by additive genetic influences (A), common (C) and unique environment (E) are shown in Fig 3. Both A and C account for a substantial proportion of EA variation in men and women in the pre-reform cohort. However, in the post-reform cohort, for males, the importance of A and E increase, while C decreases and the parameters could not be equated across cohort (χ^2^ = 9.54; *df* = 3; p = .02), indicating a significant change in the distribution of variance sources. Proportions of A, C and E remain more stable in women and the parameters could be equated across cohorts without loss of fit in the model (χ^2^ = 2.17; *df* = 3; p = .54).

Further inspection shows that the difference in males was mainly produced by the decrease of C (χ^2^ = 4.08; *df* = 1; p = .04), while equating A or E estimates across cohort did not result in a significant loss of fit. In fact, the unstandardized estimates of genetic variance were almost the same across educational systems, indicating that absolute impact of genetics on individual differences did not vary (Table 3). Table 3 also shows that the observed discrepancies between males and females are not due to differences in variance change. In both cases, total variance is reduced similarly in the post-reform cohort. However, while in males, this reduction is almost exclusively accounted for by shared environmental factors; in females, the reduction of shared environmental variance is of a lesser magnitude, but it is accompanied by a parallel reduction of genetic variance.

**Table 3:** Unstandardized estimates of variance explained by additive genetic influences (A), common environment (C) and unique environment (E) with 95% Confidence Interval, by sex and cohort.

	Pre-reform cohort (1940–1960)		Post-reform cohort (1961–1966)	
	Males	Females	Males	Females
A	.57 (.12, .91)	.46 (.14, .76)	.57 (.18, .76)	.30 (.00, .71)
C	.56 (.25, .95)	.54 (.26, .83)	.04 (.00, .31)	.38 (.00, .66)
E	.16 (.08, .31)	.15 (.08, .25)	.25 (.12, .45)	.19 (.11, .30)
Total	1.29 (1.17, 1.42)	1.14 (1.05, 1.24)	.85 (.78,.94)	.87 (.81, .94)

## Discussion

Our results provide support to the hypothesis that changes in educational policy can affect the relative weight of genetic and environmental factors on the individual differences in educational attainment (EA). We found a significantly higher heritability in post-reform compared to pre-reform male cohorts, which appears to be the result of a significantly lower impact of shared environmental factors in the cohort exposed to the more egalitarian policy. The absolute impact of genetics on EA remained the same independently of the educational conditions, but the influence of those environmental factors common to members of the same family on the advance of the individual within the educational system was much lower for the younger male cohort, thus increasing the relative weight of genetics on EA variability after reform implementation. That is, the underlying genetically determined characteristics that affect the number of years that an individual stays in the educational system, such as cognitive or personality traits, exerted the same influence before and after the educational reform. However, the environmental factors that make members of the same family similar, such as socioeconomic status, lost impact after the reform. These changes in heritability took place in the context of a generalized increase in the mean levels of EA (see Fig 1) which is the product of environmental factors. If the factors that foster EA in the population are the same for every individual, their influence in generating differences between subjects becomes less relevant. Thus, features of the reform that affected equally to every individual, such as the extension of the years of compulsory schooling, would act by reducing the variance of environmental origin. It is not possible to determine the specific role of each intervening factor but, presumably, other changes producing equality in educational opportunities, aside from the mentioned extension, would have a similar effect.

These results expand on those obtained previously [10, 11], providing more robust support to the hypothesis that introduction of equality in educational policies reduces variation due to shared-environmental factors and, consequently, increases the heritability of EA [1, 2]. Our study strengthens the given interpretation and provides further support for a social change hypothesis. The cohorts were delimitated in the basis of the exposure to different educational policies with specific characteristics, and our analysis comes from a southern European, population-based, sample with different cultural background to that of the previous studies.

In interpreting these results it must be taken into account that the 1970 reform in Spain did take place in an evolving society and other socioeconomic changes may have collaborated to the observed changes. In fact, the early 1960’s witnessed the beginning of a period of substantial economic development [32] However this reform was not just a mere consequence or by-product of economic evolution, but the beginning of a transformation which allowed major changes in the educational structure of the country. The educational reform was accomplished because of the necessity to adapt the educational system to the changing needs of society, and the gains produced in EA supported a major change in Spain’s socioeconomic configuration. Trying to determine to what extent the significant decrease we have detected in shared-environmental variance between cohorts is due to the educational reform or to the associated economic growth is speculative, but it seems reasonable to assume that the effects of economic development on EA are largely exerted through the educational system.

The changes in the heritability of EA are only significant for males, while the relative impact of genetic and environmental influences in females is not significantly different across cohorts. This difference cannot be explained by total variance disparity in EA between genders, since the reduction of such disparity between cohorts was of a similar magnitude, as illustrated by national data presented in Fig 1(B). These results are coherent with the findings of previous Scandinavian studies [10, 11] as well as with a relatively common finding of a lower heritability in females for both educational attainment and achievement [4, 12, 20, 21]. However, recent analyses of a younger Norwegian cohort found no such gender difference [8, 33]. Although no clear account has been offered yet for this effect, a tentative explanation for these sex differences in the relative importance of genetic and environmental factors in EA, in our case, could rely on a slower pace of socio-educational changes for females. That is, women could have benefited from the reform with a slight delay as compared to men, who probably had a more direct and quick access to specific areas implemented by the reform (e.g., vocational training). The lower reduction on the unstandardized estimates of C for females than for males would go in this direction, showing that familial conditions were still important in terms of EA for the former. According to Fig 1(B), females’ mean years of schooling in Spain were below that of males for the two post-war decades. Although this index started to increase for both genders after the reform (i.e. those born after 1960), it still took some years for females to reach equality in terms of number of years within the educational system. Since our younger cohort comprises only 5 years (1961–1966) it is likely that those changes had not occurred yet for females. Additionally, the geographical area where the data was collected has shown traditionally disadvantaged socio-economic indicators within Spain, and those disadvantages are more salient for women. For instance male literacy rates in 1960 were 97% in Spain and 93% in the region, while for females those percentages were 87% and 77% respectively [23]. Also in support for this argument, the higher EA heritability found in females from younger cohorts in Norway are posited to be the result of the substantial efforts made to provide equal gender opportunities in more recent times [33]. However, firm conclusions about the observed gender differences in our results, would need incorporation of a younger group of females in the sample in order to test if the reform effects in females mirrored the male pattern if a larger time period was allowed for.

When interpreting our results, we should take into account our limitation in the capacity to explain sex differences, which adds up to the one already mentioned about the impossibility for this kind of studies to isolate the effects of a specific policy within an evolving society. Additionally, we should also point out that the sample size in this study limits the power to detect the differences in the sources of variance for AE according to sex and cohort, as seen by the large confidence intervals around some of the twin correlations and parameter estimates; and that the post-reform cohort comprises a limited amount of time. A larger sample size, incorporating younger subjects, would be desirable to confirm our findings.

In summary, these results should be interpreted with caution. The very nature and characteristics of the research question, and the methodological boundaries mentioned preclude definitive conclusions. None the less, the distinctive sample of this study confers a unique opportunity to get a first glance on the effects of a specific educational reform on the heritability of EA. The hypothesis stating that more egalitarian educational policies should bear an increase in heritability by means of a reduction in shared environmental variance, has received some support previously. Our results provide further evidence for this argument and contribute to the analysis of the role of social policies on human complex behavior.
